# Correspondence between EQ-5D health state classifications and EQ VAS scores

**DOI:** 10.1186/1477-7525-6-94

**Published:** 2008-11-07

**Authors:** David K Whynes, the TOMBOLA Group

**Affiliations:** 1 School of Economics, University of Nottingham, Nottingham NG7 2RD, UK

## Abstract

**Background:**

The EQ-5D health-related quality of life instrument comprises a health state classification followed by a health evaluation using a visual analogue scale (VAS). The EQ-5D has been employed frequently in economic evaluations, yet the relationship between the two parts of the instrument remains ill-understood. In this paper, we examine the correspondence between VAS scores and health state classifications for a large sample, and identify variables which contribute to determining the VAS scores independently of the health states as classified.

**Methods:**

A UK trial of management of low-grade abnormalities detected on screening for cervical pre-cancer (TOMBOLA) provided EQ-5D data for over 3,000 women. Information on distress and multi-dimensional health locus of control had been collected using other instruments. A linear regression model was fitted, with VAS score as the dependent variable. Independent variables comprised EQ-5D health state classifications, distress, locus of control, and socio-demographic characteristics. Equivalent EQ-5D and distress data, collected at twelve months, were available for over 2,000 of the women, enabling us to predict changes in VAS score over time from changes in EQ-5D classification and distress.

**Results:**

In addition to EQ-5D health state classification, VAS score was influenced by the subject's perceived locus of control, and by her age, educational attainment, ethnic origin and smoking behaviour. Although the EQ-5D classification includes a distress dimension, the independent measure of distress was an additional determinant of VAS score. Changes in VAS score over time were explained by changes in both EQ-5D severities and distress. Women allocated to the experimental management arm of the trial reported an increase in VAS score, independently of any changes in health state and distress.

**Conclusion:**

In this sample, EQ VAS scores were predictable from the EQ-5D health state classification, although there also existed other group variables which contributed systematically and independently towards determining such scores. These variables comprised psychological disposition, socio-demographic factors such as age and education, clinically-important distress, and the clinical intervention itself.

**Trial registration:**

ISRCTN34841617

## Background

The EQ-5D is a well-established and widely-used generic instrument for assessing health-related quality of life [1]. Designed as a self-completion questionnaire, it embodies two components, a health state description followed by an evaluation. The respondent classifies his or her prevailing state of health by selecting one of three different levels of problem severity within each of five health domains. The levels are none, moderate and severe/extreme (coded 1 through 3, respectively), whilst the domains are mobility, capacity for self-care, conduct of usual activities, pain/discomfort and anxiety/depression, ordered as such. The conscious health states are therefore limited to 243 severity/domain vectors, ranging from 11111 (no problems in any domain) to 33333 (severe problems in all five domains). Having located the current health state, the respondent then evaluates his or her health using a visual analogue scale (VAS). This is a vertical, calibrated, line, bounded at 0 ("worst imaginable health state") and at 100 ("best imaginable health state"). Respondents indicate where they perceive their present state of health to lie, relative to these anchors.

Although the VAS was always integral to the EQ-5D, its role changed as the instrument evolved. The EQ-5D's descriptive system was designed to allow the reported health states to be evaluated, by assigning to each a quality or value weight (index score). Initially, the VAS was used to generate these weights; large population samples were invited to value defined states by indicating appropriate VAS positions [2]. Over time, however, the instrument's developers came to favour alternative methods of evaluating health states [3]. In the operational (self-report) version of the EQ-5D instrument, the VAS was retained to provide complementary information: "If the health status index is based on a set of weights derived from values from general population samples, this implies that the index can be regarded as a societal [*sic*] valuation of the respondent's health state, in contrast to the respondent's or patient's own assessment of his/her health state (EQ VAS scores)" [[4] p.11].

There is an extensive body of research on the use of the VAS in population studies. It is now evident, for example, that VAS-derived utility weights differ from those elicited using the time trade-off or standard gamble techniques [5,6]. The weights can vary between populations [7]. Population VAS ratings of conditions can differ from self-ratings, especially when the condition is more severe [8]. In clinical studies involving subjects actually suffering from illnesses or disabilities, EQ VAS scores have been shown to be responsive to the symptoms and severities indicated by condition-specific instruments [9-11]. In comparison with these lines of enquiry, however, relatively little attention has been paid to correspondence within the self-report instrument itself, to the relationship between the individual's EQ VAS score and his or her EQ-5D classification.

In view of the sequence of completion, the EQ VAS score relates to that which the individual thinks about the health state in which s/he has declared her/himself to be. Moving from the first to the second part of the EQ-5D questionnaire requires the subject to translate her/his description of personal well-being, represented by the extent of health problems in five dimensions, into a uni-dimensional value of health, however s/he cares to define it. Whilst we would certainly expect individuals to interpret the presence of more health problems, each of higher severity, as poorer rather than better health, the specific translation for each person must remain essentially subjective.

Comparing EQ-5D classifications with VAS scores amounts to an exploration of differential item functioning within the instrument [12]. Specifically, we hypothesise that there exist group variables which contribute systematically towards determining individuals' EQ VAS scores, independently of those individuals' health states as classified by the EQ-5D. We anticipate some degree of classification-independent variation for several reasons, the first being socio-demography. Age and education have already been offered as explanations for the diversity in EQ VAS scores assigned by the general public to nominal health classifications described, for example, as "excellent" or "fair" [13]. Material deprivation and ethnic background have been advanced as potential explanations for divergences between self-reported and actual health states in the US population [14]. Second, it is probable that evaluation is influenced by psychological disposition. Perceiving oneself to be in control of one's own health has been shown to influence positively both self-reported health status [15] and, more generally, subjective well-being [16]. Third, the VAS is continuous between 0 and 100, whereas the classification scheme offers only three choices of severity. EQ-5D subjects have reported feeling that the three-level choice is too coarse to describe their circumstances precisely [17]. Individuals with minor mobility problems only, for example, are likely to recognise the possibility of better health states, yet all might agree that the problems themselves are insufficient to merit assignment to "moderate" or "severe" in the EQ-5D's mobility domain. All these individuals would classify themselves as having no health problems, yet all would record EQ VAS scores of less than 100. That all would choose precisely the same VAS score seems improbable, especially in view of the variety of representational heuristics which individuals are known to employ [18].

## Methods

### Study sample

Our analysis used data collected during the TOMBOLA randomised controlled trial, a multi-centre UK study of the management of women recording low-grade cytological abnormalities (pre-cancers) as a result of routine cervical screening. TOMBOLA was instigated because of uncertainty over the most effective means of managing such women, the principal alternatives being immediate referral to colposcopy, with treatment if indicated, and cytological surveillance (Papanicoulou smear tests at six-monthly intervals) until the abnormality is seen to regress or progress [19]. The immediate referral of all cases had been thought unjustified until meta-analysis suggested that women with low-grade abnormalities were at risk of eventually developing invasive cancer despite continued surveillance [20]. Compared with referral, surveillance was believed to result in more defaults from follow-up and in more pre-cancers being missed [21]. On the other hand, colposcopy-for-all is the more expensive option [22] and might give rise to over-diagnosis and to unnecessary treatment and cervical damage [23].

As is required for all national trials in the UK, TOMBOLA's recruitment and analysis protocol, including the research reported in this paper, had been granted full ethical approval [24]. Cervical screening subjects are typically asymptomatic and are, on average, younger than the general population. The TOMBOLA sample was in relatively good general health, except in one respect. Having been informed of their abnormal cytology results, many of the women displayed elevated levels of anxiety and depression [25]. At the time of recruitment, TOMBOLA subjects provided basic socio-demographic information and completed an array of quality-of-life and attitude questionnaires, comprising both context-specific instruments and the EQ-5D. They were then randomised into two trial arms and managed accordingly. The control arm of the trial replicated current UK practice, namely, cytological surveillance. Those randomised to the active arm were referred immediately to colposcopy, receiving treatment as required (the current management practice for high-grade abnormalities). The majority of subjects in both arms completed a further array of questionnaires at 12 months after recruitment.

### Measures

With anxiety and distress expected to be the principal morbidity, TOMBOLA employed the Hospital Anxiety and Depression Scale (HADS) as a specific measurement instrument. The HADS was developed to identify "caseness" with respect to anxiety, mood disorders and depression in non-psychiatric settings. It has been validated as a screening tool in a clinical context and has been used as a primary instrument in investigations of both patients and populations [26,27]. The HADS assesses depression and anxiety independently on two sub-scales. Comparison of the item scores for each sub-scale with established cut-off values enables the investigator to identify possible or probable cases of anxiety or depression. TOMBOLA recruits also completed the Multi-dimensional Health Locus of Control Scale (MHLCS), an instrument which locates subjects' perceived source of control over their own health [28]. MHLCS comprises three ordinal sub-scales, each consisting of six statements. To each statement, one of six levels of agreement is assigned (scored 1 through 6), enabling summation to a sub-scale total. The Internal sub-scale assesses the extent to which the subject perceives his/her health to be under his/her own direct control. The External (or "powerful others") sub-scale assesses the perceived importance of other people, for example, physicians and family, in determining health, whilst the Chance sub-scale assesses the perceived importance of luck or fate. Each sub-scale has a range of 6-to-36, with higher values indicating stronger beliefs in that particular source of control.

The HADS, EQ-5D and MHLCS data were all scored according to the conventional algorithms. Subjects with missing HADS or EQ-5D data were excluded from the analysis. The MHLCS algorithms permit imputation when data is missing in part, although imputation was necessary only in a small number of cases (< 3 per cent). The EQ-5D index scores were derived from the current UK tariff [29,30], which has a maximum value of 1 (for health state 11111) and a minimum value of -0.59 (for 33333). Material deprivation was represented by the small-area Carstairs score, a composite measure comprising four poverty-associated variables and based on data collected during the decennial national census [31]. All such areas are ranked and divided into national quintiles, ranging from the least- to the most-deprived. Each subject was assigned to one of these quintiles, as determined by their home address.

### Analysis

We modelled EQ VAS scores using ordinary least squares linear regression. Given the hypothesis under investigation, the model contained EQ-5D health state classifications as independent variables. In addition we included, first, the HADS classification, to appraise the possibility that the EQ-5D classifications pertaining to the principal morbidity were insufficient in themselves to explain health state values. Second, we included the MHLCS scores, anticipating that individuals who believed that they controlled their own health destinies would report higher subjective values of their health state. Finally, we included a range of socio-demographic variables, with no necessary expectation of sign on the coefficients, on the basis of previous reports of associations between health values and socio-demographic factors. Carstairs scores were not included as potential explanatory variables because they proved to be collinear with the majority of individual characteristics.

To assess the stability of any relationship, we modelled changes in the EQ VAS score over the 12 months between the two questionnaire arrays using, as independent variables, changes in the EQ-5D and in the HADS classifications, plus the socio-demographic variables. We investigated the impact of one further factor in this second model, hypothesising that EQ VAS scores reported at the second round of questionnaires would have been influenced by the allocation to trial arms, for two reasons. First, recruits to clinical trials necessarily accept that they cannot pre-determine the management method to which they will be assigned, yet agreement to be randomised need not imply indifference to the randomisation outcome. It is established that a preference for the new practice under investigation (i.e. an intervention not routinely available) is a principal explanation for volunteering to participate in trials [32], whilst an unwillingness to risk randomisation away from current practice was found to be a principal explanation for refusal to participate in TOMBOLA [33]. It is therefore likely than a prior preference for the new intervention (immediate colposcopy) was widespread amongst TOMBOLA recruits. Second, by 12 months, the cervical abnormalities detected in women randomised to the colposcopy arm would have been resolved according to protocol. A proportion of women randomised to current practice, however, remained under surveillance, and the uncertainties over their abnormalities remained unresolved. We therefore judge that women randomised to the current practice arm of the trial (surveillance) might rate their health as worse, by virtue of being denied the intervention which they had sought and of failing, in some cases, to have their uncertainties resolved.

## Results

The initial analysis was based on data from the recruitment questionnaire array for 3,132 subjects. All were aged between 20 and 59 years. 53 different EQ-5D vectors were represented in this recruitment sample, although 11111 (no health problems in any of the five domains) was the most frequently cited, by 53.9 per cent of subjects. Only 3.9 per cent of subjects recorded an index score at or below 0.6, the lowest being -0.23. A further 41.8 per cent recorded scores higher than 0.6 but up to and including 0.85. The proportions of EQ VAS scores up to 60, and higher than 60 but up to and including 85, were 7.2 and 45.8 per cent, respectively. 24.9 per cent of subjects recorded scores of 90 and above, including 5.4 per cent who recorded the maximum score of 100. For those individuals recording the 11111 health state, the mean EQ VAS score was 87.0 (SD 10.7); for the remainder, it was 74.5 (SD 17.5). The index and EQ VAS scores were significantly correlated (r = 0.51, p < 0.01).

Table 1 displays the characteristics of the recruitment sample, both by Carstairs quintile and overall. Differences in sample composition as defined by Carstairs quintile were, for the categorical variables, subjected to the chi-squared test. Differences for continuous variables were subjected to one-way analysis of variance with Bonferroni adjustment. Women drawn from quintiles characterised as being less-deprived were more likely to be older, white, cohabiting, non-smoking and with formal academic qualifications. The prevalence of HADS-assessed anxiety and depression, and the likelihood of not working, increased with deprivation quintile. The MHLCS scores indicated that women from the most-deprived quintile placed more emphasis on both external factors and chance as controllers of health. Increased deprivation was associated with lower mean EQ-5D index scores and lower mean EQ VAS scores.

**Table 1:** Sample characteristics, by Carstairs quintiles

	Least-deprived			Most-deprived				
	1	2	3	4	5	Full sample	χ^2 ^or F	p =
		
Composition of sample, %	14.4	18.9	16.2	26.5	24.1	100.0		
Ethnicity, % non-white	1.6	2.4	2.4	3.5	7.3	3.7	38.4	< 0.01
Cohabiting or married, %	61.6	60.0	64.7	51.2	45.4	55.1	65.2	< 0.01
Ever had children, %	54.7	54.5	61.0	51.9	53.2	54.6	11.3	0.02
Employment, %								
Full-time	57.1	55.9	51.1	49.6	45.8	51.2	54.9	< 0.01
Part-time	23.1	22.5	27.5	21.4	21.0	22.7		
Student	8.2	7.1	6.3	11.6	11.8	9.5		
Not working	11.6	14.4	15.0	17.4	21.4	16.6		
Training and qualifications, %								
None	18.0	22.3	26.7	26.2	30.7	25.5	58.9	< 0.01
Via employment	17.3	20.5	21.2	18.0	21.1	19.6		
Up to University level	29.8	29.8	30.7	28.0	29.0	29.3		
University degree	34.9	27.4	21.4	27.8	19.1	25.6		
Current cigarette smoker, %	26.8	26.0	27.9	38.2	44.8	34.2	81.4	< 0.01
HADS anxiety, %								
Not a case	61.7	64.1	57.2	55.9	52.3	57.7	30.8	< 0.01
Possible	18.8	17.5	22.0	19.8	19.4	19.5		
Probable	19.5	18.4	20.8	24.4	28.3	22.9		
HADS depression, %								
Not a case	93.9	92.6	91.8	91.1	89.3	91.5	18.2	< 0.01
Possible	5.4	4.8	5.0	7.1	7.3	6.1		
Probable	0.7	2.6	3.2	1.8	3.4	2.4		
Mean age, years	35.0	35.5	35.1	32.3	30.7	33.4	26.6	< 0.01
Mean MHLCS score								
Internal	26.2	26.5	26.3	26.1	25.8	26.2	2.4	0.05
External	16.1	16.4	16.8	16.7	17.5	16.8	5.0	< 0.01
Chance	17.9	18.7	19.2	19.0	19.1	18.9	5.1	< 0.01
Mean EQ-5D score								
Index	0.911	0.891	0.890	0.880	0.863	0.884	6.3	< 0.01
VAS	83.6	82.0	82.6	80.6	78.6	81.1	9.5	< 0.01

All of the Table 1 variables, with the exception of the EQ-5D index score, were candidates for the first regression analysis. Age and MHLCS were entered as continuous variables, whilst the remaining variables (plus the EQ-5D classifications by severity and domain) were entered as dummies. Owing to the very small numbers of women reporting level 3 problems in the mobility, self-care and usual activities domains (n = 4, 0 and 11, respectively), those with problems at levels 2 or 3 were combined for these dimensions. The regression was estimated and re-estimated after excluding variables with insignificant coefficients, to produce the model displayed in Table 2. The signs associated with the EQ-5D domain coefficients, and the relative magnitudes associated with the severity of problem reported, are as would be expected. More severe health problems in any dimension evidently gave rise to a lower EQ VAS value for self-reported health. For any given EQ-5D health state classification, the EQ VAS score was lower if the respondent had a university degree, was a current cigarette smoker, was non-white, was likely to be anxious and/or depressed as assessed by the HADS, or located control over her health in others. The EQ VAS score was higher if the respondent was older, or located control over her health in herself.

**Table 2:** Regression, predicting VAS score

	β	T-ratio	p =
Constant	83.23	49.32	< 0.01
Age, years	0.05	2.36	0.02
University degree = 1	-1.25	-2.29	0.02
Current smoker = 1	-2.84	-5.63	< 0.01
Ethnicity, non-white = 1	-4.17	-3.35	< 0.01
HADS			
Possible anxiety = 1	-1.65	-2.61	0.01
Probable anxiety = 1	-3.22	-4.52	< 0.01
Possible depression = 1	-6.32	-5.90	< 0.01
Probable depression = 1	-5.71	-3.45	< 0.01
MHLCS			
Internal	0.29	-3.33	< 0.01
External	-0.25	-2.62	0.01
EQ-5D domain and level			
Mobility 2/3 = 1	-4.61	-6.07	< 0.01
Self-care 2/3 = 1	-6.35	-8.60	< 0.01
Usual activities 2/3 = 1	-6.77	-5.88	< 0.01
Pain/discomfort 2 = 1	-5.11	-9.19	< 0.01
Pain/discomfort 3 = 1	-12.94	-10.79	< 0.01
Anxiety/depression 2 = 1	-5.47	5.17	< 0.01
Anxiety/depression 3 = 1	-19.56	-5.91	< 0.01
Adjusted r^2^			0.32

Matched EQ-5D and HADS data over two time points (recruitment and 12 months thereafter) were available for 2,176 of the subjects. Of these, 50.6 per cent had been randomised after recruitment to immediate colposcopy, leaving the remainder to undergo cytological surveillance (current practice). The data enabled us to calculate, for each individual, (i) the change in the EQ VAS score over the period, (ii) changes in the severity of health problems in each of the five EQ-5D domains, (iii) the change in the likelihood of HADS caseness. With respect to (ii), we constructed two dummy variables for each domain, one taking the value of unity if the severity of health problem had increased (for example, a move from level 1 to level 2), the other being unity if it had decreased (for example, a move from level 3 to level 1). Likewise, with respect to (iii), dummies represented the likelihood of caseness increasing over time (for example, a move from no-case to probable anxiety) or decreasing (for example, a move from probable to possible depression). In this two-period sample, the likelihood of HADS-anxiety and HADS-depression caseness changed for 35.9 and 12.3 per cent of subjects, respectively. The EQ VAS scores changed for 85.0 per cent of subjects, with a mean fall over the period of 1.5 (SD 15.1, IQR -5 to 10, range ± 75).

Movements in the EQ-5D domains and changes in the HADS likelihood of caseness were entered into a regression model as independent variables, with the fall in EQ VAS score as the dependant variable. The socio-demographic and MHLCS variables used in the previous model were also included, as was a dummy variable representing trial randomisation. The regression was estimated and re-estimated after excluding variables with insignificant coefficients, to produce the model displayed in Table 3. The coefficients for the EQ-5D variables were as anticipated; an increased (decreased) severity of problem in any single domain contributed to a fall (rise) in the EQ VAS score from the recruitment baseline. For given changes in health state, the EQ VAS score fell (rose) if the likelihood of HADS-caseness of anxiety and/or depression increased (decreased). In the initial estimation, none of the coefficients for the socio-demographic variables had achieved significance, implying that the VAS response to changing health states was independent of such factors. For a given health state and HADS-caseness, those randomised to the immediate colposcopy arm of the trial (i.e. away from current practice) reported an increase in EQ VAS score.

**Table 3:** Regression, predicting decrease in VAS score

	β	T-ratio	p =
Constant	1.19	2.31	0.02
EQ-5D, level increases			
Mobility	6.60	3.19	< 0.01
Self-care	13.31	3.28	< 0.01
Usual activities	5.55	3.70	< 0.01
Pain/discomfort	3.70	3.62	< 0.01
Anxiety/depression	5.31	5.56	< 0.01
HADS, case more likely			
Anxiety	3.25	3.56	< 0.01
Depression	8.38	6.82	< 0.01
EQ-5D, level decreases			
Usual activities	-3.33	-2.15	0.03
Pain/discomfort	-2.84	-3.02	< 0.01
Anxiety/depression	-3.60	-3.81	< 0.01
HADS, case less likely			
Anxiety	-2.42	-3.02	< 0.01
Depression	-3.56	-2.36	0.02
Randomised to immediate colposcopy = 1	-1.50	-2.53	0.01
Adjusted r^2^			0.15

Moving between the Table 2 and the Table 3 models entailed the exclusion of 956 women from the sample. Although all of these women had supplied sufficient data at recruitment, they failed to supply EQ-5D scores or other necessary data at 12 months. To investigate selection bias, the characteristics of the excluded women were compared with the 2,176 supplying adequate data both at recruitment and at 12 months. Sample composition by Carstairs quintile differed significantly (χ^2 ^= 43.10, p < 0.01). Of the sample used in the Table 3 model, 36.2 per cent was drawn from the two least-deprived quintiles, compared with only 26.8 per cent for the excluded women. The associations between individual characteristics and deprivation levels evident in Table 1 were reproduced: excluded women, for example, were significantly more likely to be young, smokers, unemployed and uneducated. It is not evident, however, that excluding cases between the Table 2 and Table 3 models necessarily compromised the findings. First, the addition of Carstairs dummy variables to the Table 3 model produced insignificant coefficients for any (at p = 0.27 or greater), suggesting that VAS changes were independent of deprivation. Second, re-estimating the Table 2 model using the smaller, Table 3, sample, did not affect the formulation. No new variables appeared and no signs on existing variables changed, although the coefficients for age and EQ-5D mobility did become statistically insignificant.

In the light of the relatively low coefficients of determination, an analysis of residuals was conducted for each of the regressions. In each case, the scatter-plot of residuals against predicted values revealed a random pattern in the distribution of outliers, and the normal probability plot was essentially linear.

## Discussion

It appears that very few studies directly comparable to ours have been conducted. One employing the same method was based on EQ-5D data obtained from around 1,200 inhabitants of a South African suburb [34]. This study's regression model suggested that, over and above health state classification, significantly lower VAS scores were associated with the presence of disability, being older, unemployment and being in the lowest possible income band. The South African model shares three similarities with our own. First, coefficients for EQ-5D health states were significant and appropriately signed and, second, the reporting or detection of a co-morbidity (disability in the South African case, distress in ours) resulted in a lower VAS for a given EQ-5D health state. Third, economic deprivation emerged as an independent influence, explicitly in the South African model although implicitly in ours. The characteristics which predicted higher VAS scores in our case (Table 2) – being older, having a university education, not smoking and being white – were least common amongst the most deprived (Table 1). Unlike our own sample, however, the South African sample contained both males and females across the full population age range; its mean age was around 17 years higher than was ours. Our explanation of the variance in the cross-section model (Table 2) was slightly higher than that of the South African model (r^2 ^= 0.23).

Our basic approach is also comparable with that of an Israeli study of public perception of health-related quality of life [35]. Again, the sample contained both males and females from across the full population age range, the mean age being around 25 years higher than ours. Approximately 2,000 subjects were asked to classify their health using the SF-36 quality of life instrument and to value it on a numerical scale, 100 to -30, with zero indicating "dead". Values regressed on SF-36 scores and other variables indicated that higher economic status, being younger and being female were associated with a higher value for self-reported health for given SF-36 scores. The regression explained 52 percent of the variance of the VAS scores, virtually all deriving from the SF-36 domain scores. The relatively high coefficient of determination is probably accounted for by the SF-36 being a more comprehensive descriptive system in comparison with the EQ-5D. Its 36 questions combine into eight independent multi-items scales and two summary dimensions.

Turning towards explanations of particular variables in our models, predicting the sign on an age coefficient defies intuition. We note that the positive sign on our coefficient contrasts with the negative sign identified in the South African and Israeli models, although this might well result from the absence of elderly individuals in our sample. Our age coefficient pertains to a narrower age range. It is possible that the cigarette smokers valued their health states lower relative to non-smokers simply by virtue of being smokers. The messages of public health promotion initiatives over the past few decades have emphasised constantly the damage to health entailed by cigarette smoking. "Nearly all smokers regret having started smoking. Regretful smokers are those who believe themselves to be addicted. These regretful smokers report that smoking has lowered their quality of life and will continue to do so in the future. Although they are more likely to perceive that there are benefits of quitting, they have tried to quit multiple times, they have failed, and now they fear the future consequences to their health" [[36] p.349]. It follows that, if the smoker wants to give up smoking, then the best imaginable health state entails being a non-smoker which, by definition, s/he is not. Non-smokers, of course, face no such impediment when defining their best imaginable health state.

The lower value placed on health by those with a university education replicates the greater distance between index and EQ VAS score found for those with longer periods of schooling in a US study [37]. Why the possession of a university degree should influence individuals' evaluation of their own health status levels remains unclear, however. A similar comment can made with respect to ethnicity, although an ethnic influence on both classification and valuation has already been identified within the US population. In one US study, Asians were found to be significantly more likely than Whites to classify themselves as EQ-5D state 11111, even allowing for objective health conditions, education and income [38]. In another, Blacks perceived extreme health problems to be associated with less disutility than did Hispanics [39]. A Swedish study concluded that differences in self-reported health between native and immigrant populations were only partially explained by economic and psycho-social factors [40]. Cultural differences might well extend beyond non-monetary health state valuations, given that significant differences in valuations of risk reduction by ethnic background have been demonstrated in a contingent valuation study [41].

The presence of anxiety and depression effects in both the Table 2 and Table 3 models was perhaps the most surprising result, given that both types of health problem figure explicitly in the EQ-5D classification instrument. "Anxiety/depression" is one of the five named domains. Although they had been given the opportunity to record their anxiety/depression problems directly, individuals who were more likely to be suffering from HADS-anxiety and/or HADS-depression recorded EQ VAS scores disproportionately low in relation to the severity of their problems as they themselves had classified them. Changes in EQ VAS scores were determined by changes in the likelihood of HADS-identified anxiety and/or depression, in addition to any change in the assigned health state. By inference, the EQ-5D health state description system must have been inadequate to represent values of that which constituted anxiety and depression to individuals in such circumstances. In respect of our data, it might be felt that the problem arises by virtue of the absence of substantial numbers of subjects exhibiting distress and mood disorders, evidenced by a majority classifying themselves as 11111. However, the coarseness of the anxiety/depression classification has also been demonstrated for samples of patients wherein the majority were experiencing major anxiety disorders and depressive episodes [42,43].

The coefficients of determination for our regression models indicate that the model specifications leave a large part of the variance unexplained. The analysis of residuals supports the belief that the unexplained portion is attributable to randomness in individual choices. Indeed, an experiment involving the valuation of hypothetical states using VAS and time trade off methods concluded that "individual response patterns (unrelated to age or other identifiable respondent characteristics) were the main source of 'noise' in the scores" [[44] p.9]. This having been said, individual response patterns are, in principle, amenable to psychological analysis, and the inability to detect an explanation might simply point to insufficient data. Our models identified two psychological factors explaining individual responses. First, women randomised to a new, experimental, method of management recorded a smaller fall in mean EQ VAS score for a given change in health state classification. This result is consistent with our prior expectation that the self-perceived health of women undergoing a less-preferred method of management, which is, in itself, slower in resolving uncertainties, would be poorer than those undergoing the more-favoured alternative. Second, and again in keeping with our prior expectation, the quality of self-reported health for any health state was higher amongst individuals with stronger Internal, and weaker External, loci of control. Whilst it is likely that part of the variation in VAS scores is genuinely random, we would nominate personality factors, such as extroversion and conscientiousness, as strong candidates to fill at least some of the explanatory void in future research. Indeed, personality factors have been shown to be significant predictors of self-perceived health, independently of actual health problems [45].

## Conclusion

The results confirm our hypothesis that there exist group variables which contribute systematically towards determining EQ VAS scores independently of EQ-5D health state classification. In our study, these variables comprised psychological disposition, socio-demographic factors, management method and clinically-important distress.

## Competing interests

The authors declare that they have no competing interests.

## References

[B1] Rabin R, De Charro F (2001). EQ-5D: a measure of health status from the EuroQoL Group. Annals of Medicine.

[B2] Greiner W, Weijnen T, Nieuwenhuizen M, Oppe S, Badia X, Busschbach J, Buxton M, Dolan P, Kind P, Krabbe P (2003). A single European currency for EQ-5D health states. Results from a six-country study. European Journal of Health Economics.

[B3] Brooks R, with the EuroQoL Group (1996). EuroQoL: the current state of play. Health Policy.

[B4] Krabbe P, Weijnen T, Brooks R, Rabin R, De Charro F (2003). Guidelines for analysing and reporting EQ-5D outcomes. The measurement and valuation of health status using EQ-5D: a European perspective.

[B5] de Wit A, Busschbach JJV, de Charro FT (2000). Sensitivity and perspective in the valuation of health status: whose values count?. Health Economics.

[B6] Happich M, von Lengerke T (2005). Valuing the health state 'tinnitus': differences between patients and the general public. Hearing Research.

[B7] Johnson JA, Ohinmaa A, Murt B, Sintonen H, Coons SJ (2000). Comparison of Finnish and U.S.-based visual analog scale valuations of the EQ-5D measure. Medical Decision Making.

[B8] McPherson K, Myers J, Taylor WJ, McNaughton HK, Weatherall M (2004). Self-valuation and societal valuations of health state differ with disease severity in chronic and disabling conditions. Medical Care.

[B9] Sandblom G, Carlsson P, Sigsjö P, Varenhorst E (2001). Pain and health-related quality of life in a geographically defined population of men with prostate cancer. British Journal of Cancer.

[B10] Parkin D, Rice N, Jacoby A, Doughty J (2004). Use of a visual analogue scale in a daily patient diary: modelling cross-sectional time-series data on health-related quality of life. Social Science and Medicine.

[B11] Bushnell DM, Martin ML, Ricci J-F, Bracco A (2006). Performance of the EQ-5D in patients with irritable bowel syndrome. Value in Health.

[B12] Teresi JA, Fleishman JA (2007). Differential item functioning and health assessment. Quality of Life Research.

[B13] Llach XB, Herdman M, Schiaffino A (1999). Determining correspondence between scores on the EQ-5D "thermometer" and a 5-point categorical rating scale. Medical Care.

[B14] Franks P, Muennig P, Lubetkin E, Jia H (2006). The burden of disease associated with being African-American in the United States and the contribution of socio-economic status. Social Science and Medicine.

[B15] Gebhardt WA, Doef MP van der, Paul LB (2001). The Revised Health Hardiness Inventory (RHHI-24); psychometric properties and relationship with self-reported health and health behavior in two Dutch samples. Health Education Research.

[B16] DeNeve KM, Copper H (1998). The happy personality: a meta-analysis of 137 personality traits and subjective well-being. Psychological Bulletin.

[B17] Devlin NJ, Hansen P, Selai C (2004). Understanding health state valuations: a qualitative analysis of respondents' comments. Quality of Life Research.

[B18] van Osch SMC, Stiggelbout AM (2005). Understanding VAS valuations: qualitative data on the cognitive process. Quality of Life Research.

[B19] Lindeque BG (2005). Management of cervical premalignant lesions. Best Practice and Research Clinical Obstetrics and Gynaecology.

[B20] Soutter WP, Fletcher A (1994). Invasive cancer of the cervix in women with mild dyskaryosis followed up cytologically. British Medical Journal.

[B21] Flannelly G, Anderson D, Kitchener HC, Mann EMF, Campbell M, Fisher P, Walker F, Templeton AA (1994). Management of women with mild and moderate cervical dyskaryosis. British Medical Journal.

[B22] Flannelly G, Campbell MK, Meldrum P, Torgerson DJ, Templeton A, Kitchener HC (1997). Immediate colposcopy or cytological surveillance for women with mild dyskariosis: a cost effectiveness analysis. Journal of Public Health Medicine.

[B23] Jones MH (1998). The management dilemma of the mildly abnormal smear: fact or fiction?. Annals of the Academy of Medicine of Singapore.

[B24] Cotton SC, Sharp L, Little J, Duncan I, Alexander L, Cruickshank ME, Gray NM, Jenkins D, Philips Z, Robertson A (2006). Trial of management of borderline and other low-grade abnormal smears (TOMBOLA): trial design. Contemporary Clinical Trials.

[B25] Gray NM, Sharp L, Cotton SC, Masson LF, Little J, Walker LG, Avis M, Philips Z, Russell I, Whynes D (2006). Psychological effects of a low-grade abnormal cervical smear test result: anxiety and associated factors. British Journal of Cancer.

[B26] Herrmann C (1997). International experiences with the Hospital Anxiety and Depression Scale: a review of validation data and clinical results. Journal of Psychosomatic Research.

[B27] Bjelland I, Dahl AA, Haug TT, Neckelmann D (2002). The validity of the Hospital Anxiety and Depression Scale: an updated literature review. Journal of Psychosomatic Research.

[B28] Wallston KA (2005). The validity of the Multidimensional Health Locus of Control Scales. Journal of Health Psychology.

[B29] Dolan P, Gudex C, Kind P, Williams A (1996). The time trade-off method: results from a general population study. Health Economics.

[B30] Dolan P (1997). Modeling valuations for EuroQol health states. Medical Care.

[B31] Morgan O, Baker A (2006). Measuring deprivation in England and Wales using 2001 Carstairs scores. Health Statistics Quarterly.

[B32] King M, Nazareth I, Lampe F, Bower P, Chandler M, Morou M, Sibbald B, Lai R (2005). Impact of participant and physician intervention preferences on randomized trials: a systematic review. Journal of the American Medical Association.

[B33] Sharp L, Cotton SC, Alexander L, Williams E, Gray NM, Reid JM, on behalf of the TOMBOLA Group (2006). Reasons for participation and non-participation in a randomised controlled trial: postal questionnaire surveys of women eligible for TOMBOLA (Trial Of Management of Borderline and Other Low-grade Abnormal smears). Clinical Trials.

[B34] Jelsma J, Ferguson G (2004). The determinants of self-reported health-related quality of life in a culturally and socially diverse South African community. Bulletin of the World Health Organization.

[B35] Mozes B, Maor Y, Shmueli A (1999). Do we know what global ratings of health-related quality of life measure?. Quality of Life Research.

[B36] Fong GT, Hammond D, Laux FL, Zanna MP, Cummings KM, Borland R, Ross H (2004). The near-universal experience of regret among smokers in four countries: findings from the International Tobacco Control Policy Evaluation Survey. Nicotine and Tobacco Research.

[B37] Franks P, Lubetkin EI, Melnikov J (2007). Do personal and societal preferences differ by socio-demographic group?. Health Economics.

[B38] Fu AZ, Kattan MW (2006). Racial and ethnic differences in preference-based health status measure. Current Medical Research and Opinion.

[B39] Shaw JW, Johnson JA, Chen S, Levin JR, Coons SJ (2007). Racial/ethnic differences in preferences for the EQ-5D health states: results from the US valuation study. Journal of Clinical Epidemiology.

[B40] Lindström M, Sundquist J, Östergren P-O (2001). Ethnic differences in self reported health in Malmö in southern Sweden. Journal of Epidemiology and Community Health.

[B41] Whynes DK, Frew EJ, Philips ZN, Covey J, Smith RD (2007). On the numerical forms of contingent valuation responses. Journal of Economic Psychology.

[B42] Supina AL, Johnson JA, Patten SB, Williams JVA, Maxwell CJ (2007). The usefulness of the EQ-5D in differentiating among persons with major depressive episode and anxiety. Quality of Life Research.

[B43] Günther OH, Roick C, Angermeyer MC, König H-H (2008). The responsiveness of EQ-5D utility scores in patients with depression: a comparison with instruments measuring quality of life, psychopathology and social functioning. Journal of Affective Disorders.

[B44] Essink-Bot MLE, Stuifbergen MC, Meerding WJ, Looman CWN, Bonsel GJ, the VOTE group (2007). Individual differences in the use of the response scale determine valuations of hypothetical health states: an empirical study. BMC Health Services Research.

[B45] Goodwin R, Engstrom G (2002). Personality and the perception of health in the general population. Psychological Medicine.

